# Impacts of habitat connectivity on grassland arthropod metacommunity structure: A field‐based experimental test of theory

**DOI:** 10.1002/ece3.10686

**Published:** 2023-11-07

**Authors:** Franklin Bertellotti, Nathalie R. Sommer, Oswald J. Schmitz, Matthew A. McCary

**Affiliations:** ^1^ School of the Environment Yale University New Haven Connecticut USA; ^2^ Department of Biosciences Rice University Houston Texas USA

**Keywords:** arthropods, conservation management, ecological restoration, grasslands, habitat connectivity, metacommunity structure

## Abstract

Metacommunity theory has advanced scientific understanding of how species interactions and spatial processes influence patterns of biodiversity and community structure across landscapes. While the central tenets of metacommunity theory have been promoted as pivotal considerations for conservation management, few field experiments have tested the validity of metacommunity predictions. Here, we tested one key prediction of metacommunity theory—that decreasing habitat connectivity should erode metacommunity structure by hindering species movement between patches. For 2 years, we manipulated an experimental old‐field grassland ecosystem via mowing to represent four levels of habitat connectivity: (1) open control, (2) full connectivity, (3) partial connectivity, and (4) no connectivity. Within each treatment plot (10 × 10 m, *n* = 4 replicates), we measured the abundance and diversity (i.e., alpha and beta) of both flying and ground arthropods using sticky and pitfall traps, respectively. We found that the abundance and diversity of highly mobile flying arthropods were unaffected by habitat connectivity, whereas less mobile ground arthropods were highly impacted. The mean total abundance of ground arthropods was 2.5× and 2× higher in the control and partially connected plots compared to isolated patches, respectively. We also reveal that habitat connectivity affected the trophic interactions of ground arthropods, with predators (e.g., wolf spiders, ground spiders) being highly positively correlated with micro‐detritivores (springtails, mites) but not macro‐detritivores (millipedes, isopods) as habitat connectivity increased. Together these findings indicate that changes in habitat connectivity can alter the metacommunity structure for less mobile organisms such as ground arthropods. Because of their essential roles in terrestrial ecosystem functioning and services, we recommend that conservationists, restoration practitioners, and land managers include principles of habitat connectivity for ground arthropods when designing biodiversity management programs.

## Graphical abstract

Habitat connectivity alters the metacommunity structure of ground arthropods, whereby completely isolated habitats have a different composition compared to more‐connected plots. Thus, conservation biologists should incorporate principles of metacommunity theory when designing biodiversity management programs.

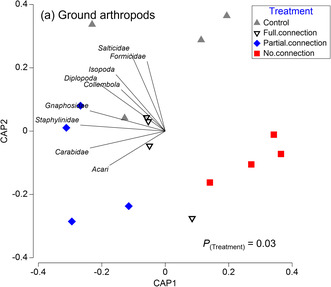

## 1 INTRODUCTION

Metacommunity theory has been central to understanding how spatial processes and species interactions determine patterns of biodiversity and community structure across landscapes (Dorazio et al., 2010; Guzman et al., 2019; Holyoak et al., 2005; Leibold et al., 2004; Mouquet & Loreau, 2003; Wilson, 1992). It has gained considerable traction because it resonates with our intuitive understanding of how species respond to landscape configuration (Collinge, 2010; Swan & Brown, 2017). For example, freshwater ponds, streams, and lakes represent natural, discrete patches within a terrestrial landscape matrix, leading to characteristic patterns of local and regional species abundances, interactions, and ecosystem functioning (de Meutter et al., 2007; Gansfort et al., 2020; Heino, 2005). Human activities have also artificially imposed spatial structure onto many terrestrial landscapes by fragmenting formerly continuous habitats into discrete patches (Gauthier et al., 2021; Johnson et al., 2013). This has transformed species assemblages and their associated functioning owing to the differential abilities of species to persist within patches of a particular size and to move among them (Fahrig, 2003). Hence, metacommunity theory can have profound value in informing the conservation of biodiversity (Chase et al., 2020; Collinge, 2010; Economo, 2011).

Yet, despite the proliferation of metacommunity research, few empirical studies have tested it (Schmera et al., 2018). Those studies that have are often observational across gradients of fragmentation; even fewer available experimental studies have been conducted in the laboratory or in artificial mesocosms (e.g., Dorazio et al., 2010; Driscoll & Lindenmayer, 2009; Gawecka & Bascompte, 2021; Logue et al., 2011; López‐Delgado et al., 2019; Pillai et al., 2011). Experiments in open field environments with natural ecological communities are thus needed to explore the efficacy of metacommunity predictions (da Silva et al., 2021), especially if metacommunity theory is to be enlisted to inform conservation (Chase et al., 2020; Gounand et al., 2018; Heino et al., 2021).

Here, we report on a field experiment that tests a key hypothesis with implications for biodiversity conservation across landscapes. The hypothesis holds that decreasing habitat connectivity via fragmentation among a network of patches should erode metacommunity structure by hindering the ability of species to move between patches (Gawecka & Bascompte, 2021; Swan & Brown, 2017). Metacommunities are a set of local communities that are linked by among‐patch dispersal of multiple interacting species at the landscape scale (Leibold et al., 2004). Hence, metacommunity structure is highly dependent on the movement of individuals of different species (specifically the degree of mobility) between habitat patches (Mouquet & Loreau, 2003). For example, large predators often move more effectively than their smaller prey and will disperse to other high‐quality patches to avoid starvation. Such movement could shift metacommunity structure by intensifying top‐down pressure in the new patch while alleviating top‐down pressure in the vacated patch (McCann et al., 2005). However, if connections between habitats are lost, predators can reshape the metacommunity structure by causing local prey extinctions via overhunting (Orrock et al., 2008). Thus, the loss of habitat connectivity between patches within a metacommunity network should inhibit the movement and exchange of species, leading to a reduction of diversity at the local (i.e., alpha diversity) and regional scale (i.e., beta diversity).

One group of organisms that is highly amenable to empirically testing the effects of connectivity on metacommunity structure is terrestrial arthropods (Braaker et al., 2014; McCary et al., 2018). Terrestrial arthropods make good candidates for several reasons. First, they are relatively small‐sized, hyper‐diverse, found in all terrestrial ecosystems, and have a limited extent of spatial movement (Bardgett & van der Putten, 2014; Coleman & Crossley, 2003; Wardle et al., 2004). Terrestrial arthropods are also sensitive to both local and landscape‐scale factors (Dauber et al., 2005; McCary et al., 2015) and occupy different trophic levels (Bardgett et al., 2005). Furthermore, they are relatively easy to sample passively via pitfall and sticky traps, allowing for a standardized, inexpensive method to monitor their movement and community composition (Thomson et al., 2004; Woodcock, 2005). All of these features allow for tractable, yet realistic, experiments within small landscape extents that can provide generalizable insights.

We capitalized on a further feature of terrestrial arthropods, namely their wide range of mobility between arthropod groups, to evaluate a second hypothesis of metacommunity theory. This hypothesis states that the importance of connectivity will vary with the mobility of species. Ground arthropods (e.g., springtails, mites, ground beetles, etc.) are less mobile and mostly relegated to the soil surface without the ability to fly (Coleman & Crossley, 2003). Some estimates indicate that ground arthropods spend their entire life cycle within just a few square meters (Coleman & Hendrix, 2000), making them highly sensitive to changes in the local environment. Alternatively, flying arthropods (butterflies, flies, moths) can be highly mobile for their small size, with some species able to move several kilometers in 1 day (Kuussaari et al., 2014). We thus evaluated how habitat connectivity might differentially affect metacommunity structure of these different mobility types of arthropods. We expected that the metacommunity structure of ground arthropods would be more sensitive to habitat connectivity than the structure of flying arthropods.

Our study asks three questions: (1) How does habitat connectivity influence the abundance and diversity (i.e., alpha and beta diversity) of terrestrial arthropods? (2) How does the level of mobility affect arthropod metacommunity structure? (3) What are the effects of habitat connectivity on arthropod trophic interactions? We manipulated the habitat structure of an old‐field grassland by mowing patches of variable connectivity, ranging from full habitat connectivity to completely isolated patches. We compare the metacommunity structure based on the level of mobility by sampling both ground and flying arthropods.

## 2 METHODS

### 2.1 Study site

We conducted the experiment in an old‐field grassland at the Yale‐Myers Research Forest in northeastern Connecticut, USA. Dominant grasses at this site include *Poa pratensis* and *Phleum pratense*, while herbs include *Trifolium repens*, *Potentilla simplex*, *Solidago rugosa*, *Solidago altissima*, *Daucus carota*, and *Asclepias syriaca*. There is also an abundance of arthropods with varying levels of mobility (Schmitz, 2008). Major herbivores include a sapsucking guild consisting of the grass‐specialist plant bug *Leptopterna dolobrata*, planthoppers *Campylenchia latipes* and *Stichtocephala festina*, generalist pentatomid *Acrosternum hilare*, generalist spittlebug *Philaenus spumaris*, Solidago specialist *Lopidea media* and *Lygaeus kalmia* (Schmitz, 2008). Predators in this system include a variety of hunting and sit‐and‐wait spiders, including *Rabidosa rabida*, *Pisaurina mira*, *Misumena vatia*, and *Phidippus clarus*. Such species can be broadly arranged into two groups: highly mobile flying arthropods (e.g., Lepidoptera, Diptera) and low‐mobility ground arthropods (e.g., Acari, Araneae, Collembola, Isopoda). While we recognize these are not perfect categories for describing arthropod mobility, it is a safe assumption that arthropods that primarily fly are more able to disperse long distances than ground‐dwelling arthropods.

### 2.2 Experimental design

The experiment began in June 2020 and continued into September 2021 to allow for any potential transient dynamics due to the initial manipulation to stabilize. We created an experimental metacommunity network by mowing areas in the field to create a fragmented patch landscape of plant communities, which were connected to varying degrees via “corridors” of un‐mowed vegetation linking patches together. Here, we used a Briggs and Stratton 55‐cm gas lawnmower (Home Depot, Atlanta, Georgia, USA) to cut the vegetation down to the ground surface (<3 cm stubble height), creating a harsh matrix compared to the adjacent grassland vegetation (>1 m in height). The experiment had four treatments (i.e., four levels of metacommunity structure): (1) an un‐mowed control to represent an unfragmented landscape and mowed (fragmented landscape) treatments with (2) full connectivity of patches, (3) partial connectivity of patches, and (4) no connection of patches (Appendix S1: Figure S1). While the main goal of this experiment was to test connectivity effects on arthropod metacommunity structure, we acknowledge that is impossible to disentangle the disturbance effect of mowing from the different levels of habitat connectivity (i.e., the less‐connected treatments also experienced higher levels of mowing disturbance). However, the less‐connected habitats occurring in natural landscapes are likely due to extreme disturbance events (e.g., habitat destruction and fragmentation), so our experimental design reflects a real‐world test of how habitat connectivity impacts metacommunity structure.

The experiment was arrayed as a randomized block design, with each treatment being replicated four times along a north–south axis at the research site. Each replicate encompassed a 10 × 10 m area containing four 2 m^2^ patches spaced 0.5 m apart. Corridors connecting the patches were 1 m wide and mowed every 3–4 weeks during the growing season to maintain the connectivity treatments. In 2020 and 2021, each of the 64 experimental patches was sampled for ground and flying arthropods. Due to the relatively large area needed for each plot (i.e., 10 m^2^ in size plus the space needed between plots) and finite space at the research site, 4 replicates per treatment was the maximum number that could reasonably fit at the site. However, despite the limited replication, we were still able to detect significant treatment differences (see details below).

### 2.3 Arthropod sampling

Ground arthropods were sampled using pitfall traps (Woodcock, 2005). For pitfall trapping, the number of arthropods collected in a trap reflects both the mobility of the organisms and their population density, generating a composite index called “activity‐density” (Southwood, 1978). We installed pitfall traps (5 cm wide and deep) in the middle of each of the 64 patches, as well as the intersections between patches, with all traps forming a 3 × 3 grid (Figure S1). Each trap (*n* = 144 traps) was filled with 250 mL of a 50:50 mix of propylene glycol and water to serve as a preservative and killing agent. Clear plastic covers were placed over each trap to exclude rain and debris. Pitfall traps were left open for 10 days. After 10 days, arthropods were collected from the traps, poured into Ziploc™ bags, and transported to the laboratory. Once at the lab, arthropods from each bag were washed and identified under 10–40× magnification to the lowest taxonomic level possible (either order, suborder, or family, depending on the observed traits). Ground arthropod abundance was calculated as the number of arthropods collected per trap divided by the number of days that the trap was active (activity‐density).

Because flight‐bearing arthropods are hypothesized to be less affected by habitat connectivity at the scale of our study, we also sampled flying arthropods. We used standard yellow sticky cards (Böckmann et al., 2021; Heinz et al., 1992) that were 20 × 15 cm in size and placed them 1 m off the ground in a 3 × 3‐grid design within each treatment replicate (*n* = 144 total traps) to capture flying arthropods, mirroring the same design as the pitfall traps. Traps were arrayed along a north–south axis. All traps were installed in July 2020 and 2021 and deployed for 10 days. Eligible arthropods were then identified to the lowest taxonomic level (either order, suborder, or family depending on relevant traits). Though the sticky traps caught some non‐flying arthropods (e.g., spiders and ants), they are not an appropriate method for assessing the abundance and diversity of non‐flying arthropods; thus, they were omitted from the final dataset.

### 2.4 Analysis

We employed three statistical approaches to examine how habitat connectivity affects arthropod abundance and diversity for both ground and flying arthropods, as well as their trophic interactions. First, we used Simpson's diversity to determine how arthropod alpha diversity differed according to habitat connectivity. Next, we investigated how habitat connectivity affects arthropod community composition (i.e., beta diversity) using Bray–Curtis similarity. This multivariate metric encompasses both taxonomic identities and their abundances and is reported to be more sensitive than univariate richness estimates (Avolio et al., 2022; Komatsu et al., 2019). Lastly, we used structural equation modeling (SEM; Grace, 2006) to distinguish how habitat connectivity affects the direct and indirect trophic interactions between micro‐arthropods, macro‐arthropods, and predators.

#### 2.4.1 Q1: Arthropod abundance and alpha diversity

We used linear mixed‐effects models (LMMs) to examine how our manipulation affected the abundance and diversity of ground and flying arthropods as a function of fixed‐factors habitat connectivity and year (2020 and 2021). Random effects in the mixed model included the experimental blocks to account for variability across plots. Ground and flying arthropod abundances were log‐transformed prior to analysis to satisfy assumptions of normality. LMMs were fit using the “lme4” package in R version 4.0.3 (Bates et al., 2015; R Development Core Team, 2022). Kenward‐Roger approximations for degrees of freedom were used to calculate *p*‐values (Type III SS) using the “lmerTest” R package version 4.0.4 (Kuznetsova et al., 2017). Tukey's HSD post hoc comparisons were also used to examine treatment comparisons using the “emmeans” R package version 4.0.5 (Lenth, 2018). Simpson's diversity metric was calculated using the “vegan” R package version 4.0.4 (Oksanen et al., 2013).

#### 2.4.2 Q2: Community composition (i.e., beta diversity)

To evaluate how habitat connectivity impacted arthropod community composition (i.e., beta diversity), we performed a permutational analysis of variance (PERMANOVA; 9999 permutations; Type III SS) (Anderson et al., 2008) using habitat connectivity and year (2020 and 2021) as fixed factors and block as a random effect (Anderson et al., 2008). Before the analysis, we averaged arthropod abundance in each plot and log‐transformed the data with singletons removed to meet assumptions of normality. We then calculated a Bray–Curtis similarity distance matrix to estimate beta diversity. To visualize differences in beta diversity as a function of habitat connectivity, we performed a Canonical Analysis of Principal Coordinates (CAP) ordination when there was a significant treatment effect, or a Principal Coordinate Analysis (PCO) when non‐significant treatment effects occurred (Anderson et al., 2008). Vector overlays were used to show which arthropod taxon was associated with each treatment. We present vector overlays with Pearson's correlation coefficients to show the relationship between arthropod taxa and the two ordination axes.

#### 2.4.3 Q3: Trophic interactions

To understand how habitat connectivity affected trophic interactions, particularly for ground arthropods, we employed piecewise structural equation models (SEMs) (Lefcheck, 2016). Piecewise SEMs can simultaneously evaluate multiple hypotheses and are useful for quantifying direct and indirect effects via joining several linear mixed‐effects models into a single global SEM. This allows complex data structures and random effects to be tested, where a set of individual equations are tested separately and then later combined to generate inferences about the full SEM. Thus, piecewise SEMs can also uncover important direct and indirect effects that single equations cannot generate on their own (Lefcheck, 2016).

Because we hypothesized a monotonic response of arthropod responses with increasing connectivity, we modeled habitat connectivity as a fixed‐factor continuous variable (i.e., 1–4), with 1 representing low connectivity (i.e., no connection) to 4 representing high connectivity (i.e., control). We included random effects with trap position nested in blocks to take advantage of the full dataset and utilize more data points per plot instead of averages. The response variables included in the SEM were the abundance of less mobile micro‐detritivores (<2 mm), highly mobile macro‐detritivores (≥2 mm), and highly mobile predators (e.g., wolf spiders, ground spiders). We used Shipley's tests of d‐separation to examine overall model fit, where *p*‐values were derived via Fisher's C test statistic (Borenstein et al., 2009). *p*‐values calculated from Fisher's C test statistic denote adequate model fits with *p* > .05. To determine the best‐fit model, we used the lowest AIC values where the best model required the primary pathways but also >2 AIC units lower than other competing SEMs. All data were log‐transformed prior to analysis, and the R package “piecewiseSEM” version 2.1.0 was used to perform the SEM analysis (Lefcheck, 2016; R Development Core Team, 2022).

## 3 RESULTS

Initial linear mixed‐effects models and PERMANOVAs revealed marginal treatment by year interactions (.05 < *p* ≤ .1) for ground arthropod abundance and beta diversity (Table S1), so we performed tests for each year separately (including the flying arthropods to mirror the analysis). Furthermore, because the experiment commenced in June 2020 and some time was needed to stabilize transient dynamics induced by mowing, we did not expect treatment differences in the first year of the experiment. Thus, we only report the results for the second year of the experiment (2021). However, we provide the full treatment effects on abundance and diversity (alpha and beta) of ground and flying arthropods in 2020 (the initial year of the experiment) in the supplemental materials (Table S2a,b); we did not find any treatment differences across any of these response variables in 2020.

**FIGURE 1:**
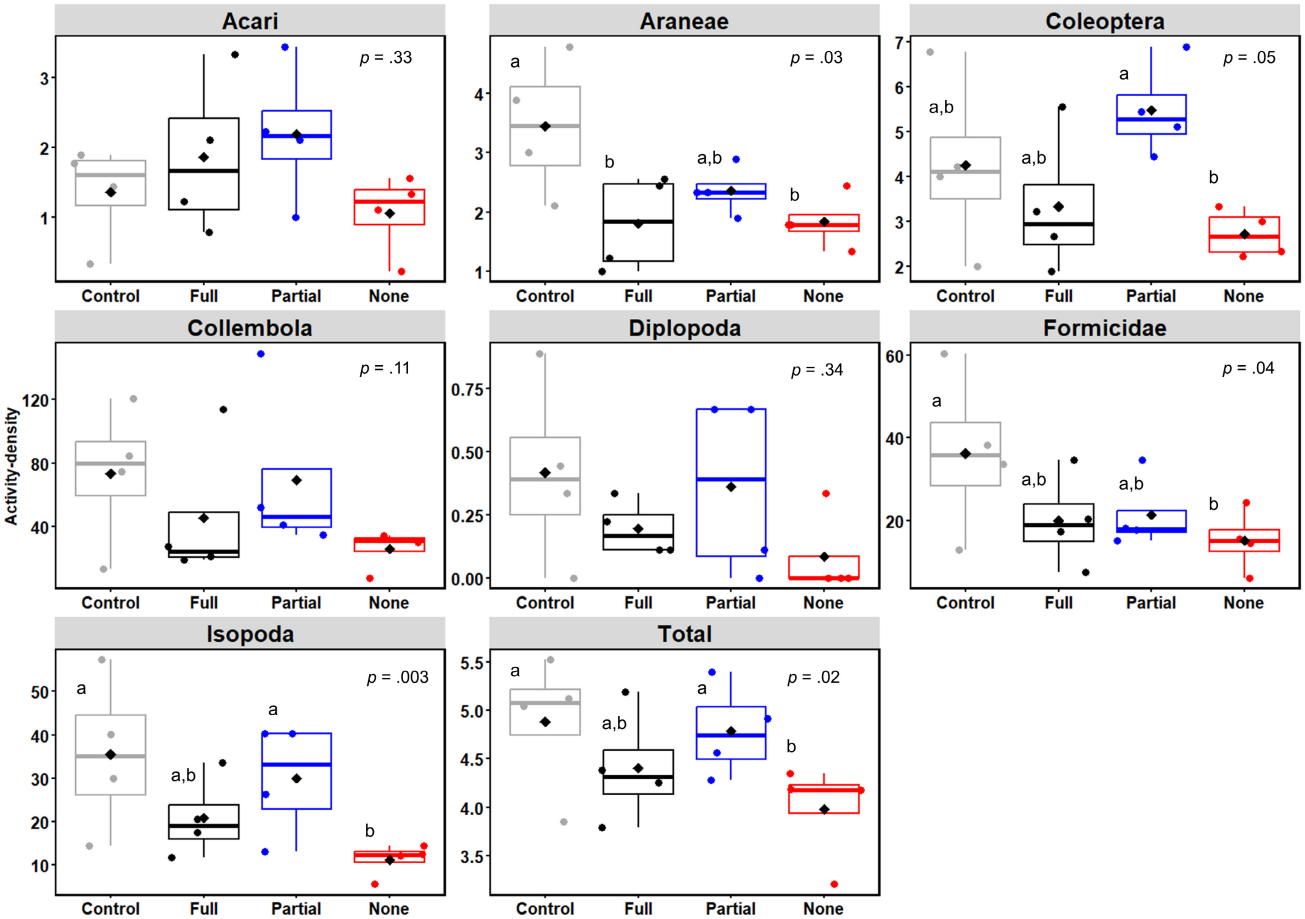
The relationship between habitat connectivity and individual ground arthropod taxa for 2021. Different letters indicate *p* < .05 using Tukey's HSD posthoc comparisons. For all boxplots, the top and bottom of the boxes indicate the first and third quartiles, with the center line denoting the median; the whiskers show 1.5 times the interquartile range. The diamond symbols are treatment means for 2021.

In the second year of the experiment (2021), we found no significant effects of habitat connectivity on arthropod alpha diversity for ground arthropods (LMM; *F*_3,9_ = 0.726, *p* = .56) or flying arthropods (*F*_3,9_ = 0.39, *p* = .76). However, activity‐density for most ground arthropods was significantly affected by habitat connectivity (Figure 1), whereas the flying arthropods were completely unaffected (Figures S2 and S3). Ground arthropods such as spiders (Araneae), beetles (Coleoptera), ants (Formicidae), and woodlice (Isopoda) were all more abundant in the control plots than in the no‐connection plots (Figure 1). In particular, spiders (LMM; *F*_3,9_ = 4.00, *p* = .03), beetles (*F*_3,9_ = 3.79, *p* = .05), ants (*F*_3,9_ = 4.35, *p* = .04), and woodlice (*F*_3,9_ = 9.41, *p* = .003) were respectively 2×, 1.6×, 2.4×, and 3× more abundant in the control plots compared to the no‐connection plots. The differences between full, partial, and no‐connection plots varied across these taxonomic groups, rendering it difficult to draw consistent conclusions. However, in general, the full‐ or partial‐connection plots appear to have higher mean values of spiders, beetles, ants, and woodlice than the no‐connection plots (Figure 1). In contrast, arthropod groups such as mites (Acari), springtails (Collembola), and millipedes (Diplopoda) did not appear sensitive to habitat connectivity, with neither group showing abundance responses to the experimental treatments (Figure 1).

**FIGURE 2:**
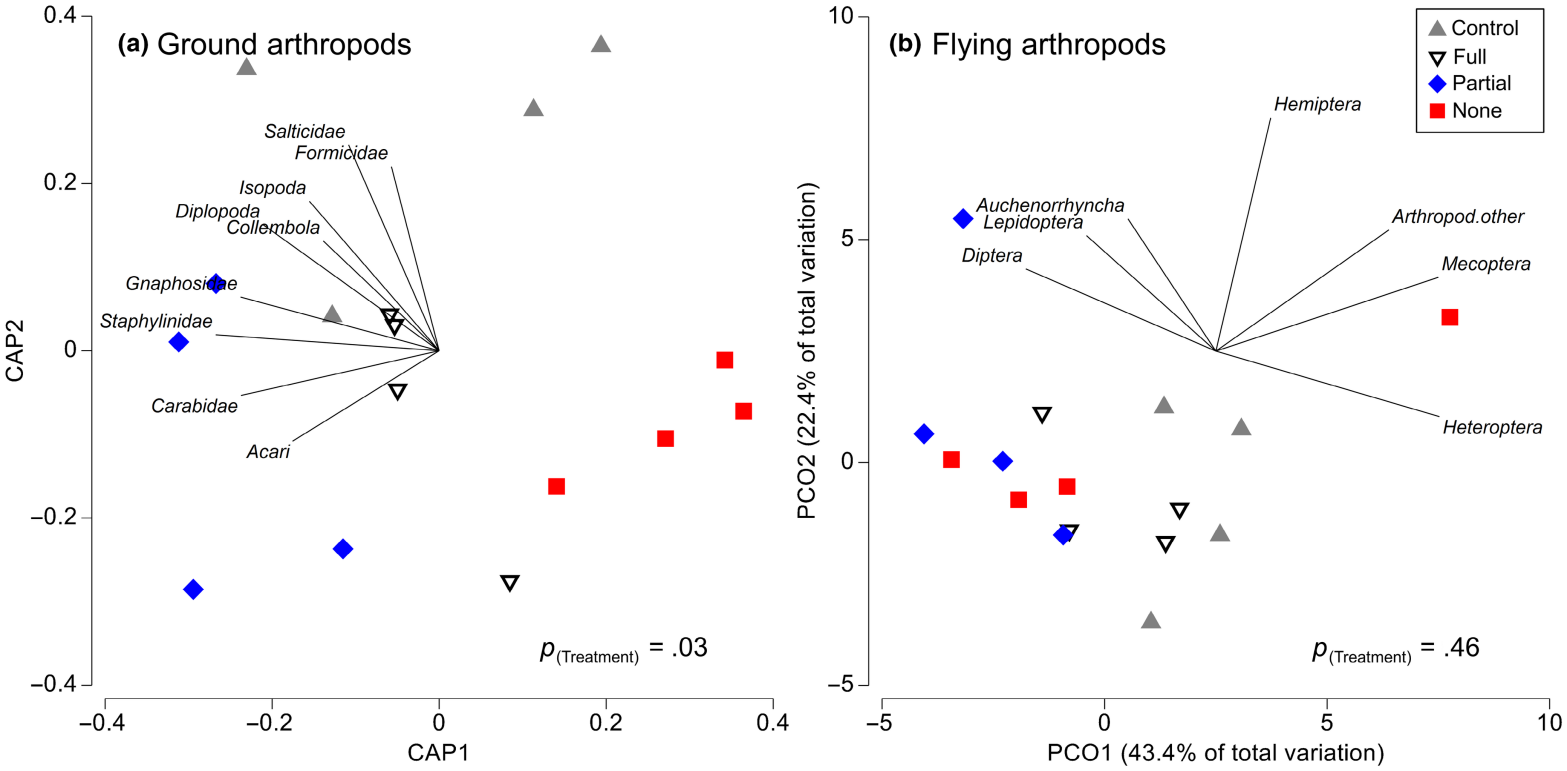
The effects of grassland connectivity on arthropod composition (i.e., beta diversity) for ground arthropods (a) and flying arthropods (b) in 2021. Ordination bi‐plots show arthropod data based on a Bray–Curtis similarity matrix. Each symbol on the ordination plot represents communities for one of the 16 experimental plots. The length and direction of vector overlays denote the strength of the relationship (Pearson's correlation coefficient with *R* ^2^ > .55) between the ordination axes and the associated arthropod taxon.

Ground arthropod beta diversity responded strongly to habitat connectivity (PERMANOVA, *Pseudo‐F*_3,9_ = 2.36, *p* = .03) in 2021, with follow‐up pairwise comparisons revealing that the no‐connection treatment was significantly different compared to the control and partial‐connection treatments (Table S3). When examining the CAP ordination for ground arthropods, the no‐connection plots clustered in the bottom right corner of the ordination plot, indicating a distinct community from the rest of the other treatments except for the full‐connection plots (Figure 2a). The full and partial‐connection plots clustered on the left side of the first axis (i.e., CAP1), suggesting those communities are distinct from the no‐connection plots. Furthermore, when examining the vector overlays, all of the major arthropod groups were highly correlated with the left side of CAP1, indicating those arthropod groups were generally more abundant in the control, partial, and full‐connection plots compared to the no‐connection plots (Figure 2a). Notably, there were no arthropod groups that correlated with the no‐connection plots. As above, we found that the composition of flying arthropods was unaffected by habitat connectivity (*Pseudo‐F*_3,9_ = 1.02, *p* = .46) (Figure 2b), with no changes in the main groups across any experimental treatment (Table S2b).

**FIGURE 3:**
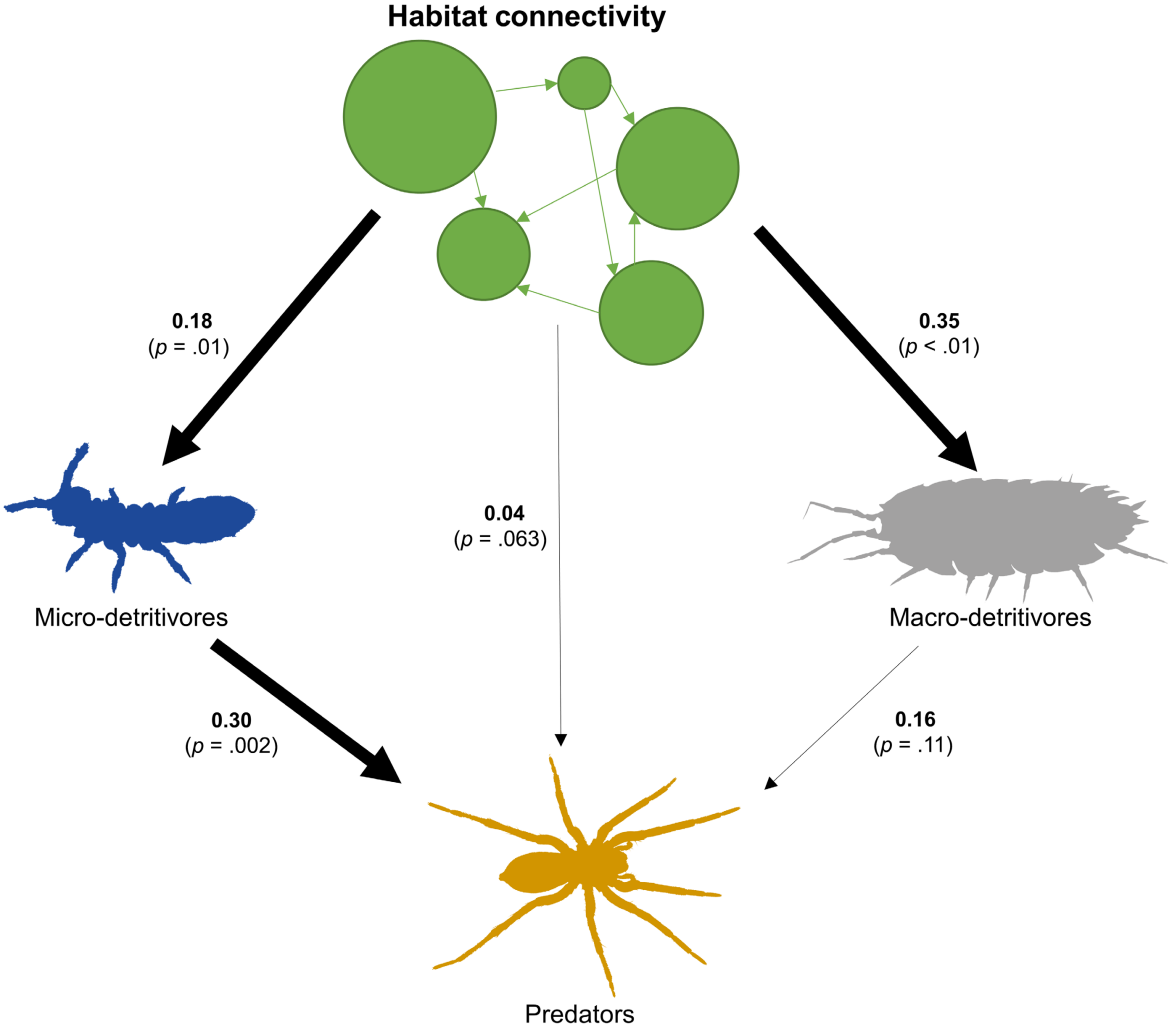
The structural equation model showing the effects of habitat connectivity on micro‐detritivores, macro‐detritivores, and predators in 2021. The bolded numbers next to the arrows are standardized coefficients, and the *p*‐values provide the strength of evidence of a given path. Lastly, the arrow's thickness denotes the strength of the relationship, with the thick arrows representing path coefficients with *p* < .05.

Our a priori SEM was an adequate fit for the data (Fisher's C = 0.00, *p* > .9, Figure 3). The SEM indicated that increasing habitat connectivity positively influenced both micro‐detritivores (0.18 [standardized coefficient], *p* = .01) and macro‐detritivores (0.35, *p* < .001) while having no effect on predators (0.04, *p* = .63). Micro‐detritivores, in turn, had a strong positive influence on arthropod predators (0.30, *p* = .002), indicating that habitat connectivity has an indirect positive effect on predators via micro‐detritivore populations. However, we found no relationship between macro‐detritivores and predators (0.16, *p* = .11). This SEM explained 29% of the variation in micro‐detritivores, 28% for macro‐detritivores, and 29% for arthropod predators.

## 4 DISCUSSION

Metacommunity theory has been promoted to improve scientific understanding of the potential emergent consequences of landscape changes, with the aim to inform biodiversity conservation (Chase et al., 2020; Economo, 2011). Yet, despite its central importance in ecology and conservation, few field experiments have tested its validity (Braaker et al., 2014; Dorazio et al., 2010; Logue et al., 2011; Pillai et al., 2011). We addressed this key knowledge gap by evaluating how the abundance and diversity (i.e., alpha and beta) of terrestrial arthropods were impacted by habitat connectivity in an experimental old‐field. Our findings show that decreasing connectivity can negatively affect arthropod metacommunity structure. The response of arthropods was partially consistent with the first hypothesis from metacommunity theory that reducing habitat connectivity will lead to a lower abundance and altered diversity patterns of ground arthropods. Moreover, arthropod responses were fully consistent with the second hypothesis from metacommunity theory, that mobility influences the sensitivity of species to habitat connectivity, thereby shaping metacommunity structure. Lastly, we reveal that habitat connectivity influences trophic interactions between functional groups of species within the metacommunity. Together our findings indicate that metacommunity theory performs well at predicting how ground arthropod communities would respond to habitat connectivity across fragmented landscapes.

Our experiment revealed a key feature of metacommunity structure, which is that decreasing habitat connectivity does indeed lead to lower abundances of ground arthropods compared to the highly connected plots by the end of the experiment (i.e., 2021). But its support varied with species mobility. Decreasing habitat connectivity reduced the abundance of less mobile arthropods (i.e., ground arthropods); however, it did not affect their overall alpha diversity among the different metacommunity structures. The differences in abundance were observed for many ground arthropods (except for mites, springtails, and millipedes), as well as the cumulative abundance for all ground arthropods. While some ground arthropods can traverse harsh terrains (Bang & Faeth, 2011), most will not attempt to cross matrices that are different from their known habitat (Moir et al., 2005; Pedley & Dolman, 2020; Vergnes et al., 2014), thereby minimizing the movement of arthropods into and out of isolated patches. In our study, the more‐connected patches appear to be replenished by surrounding source habitats more so than the isolated patches. Thus, our results for the ground arthropods support other research showing that habitat connectivity can positively influence the abundance of organisms and composition of species (Gawecka & Bascompte, 2021; Kuussaari et al., 2014; Swan & Brown, 2017).

At the beta diversity level, we found that habitat connectivity dramatically altered the composition of ground arthropods. By the end of the experiment, the no‐connection plots had a severely different community composition compared to the control and partially connected treatments. This finding indicates that the isolated patches harbored fewer ground arthropods overall but were also composed of a different community, suggesting possible environmental filtering of some species. Long‐term isolation of those plots is, therefore, likely to decrease ecosystem functionality with fewer detritivores to decompose litter and organic matter. Furthermore, we found that several arthropods were more associated with the partially connected plots, perhaps indicating an affinity for moderately disturbed habitats. For example, our CAP ordination plot shows that ground beetles (Carabidae) associate more with the partially connected plots and less with the control plots. Ground beetles have the ability to fly, and several studies indicate their abundance can be elevated in areas of high fragmentation or disturbance (Fujita et al., 2008; Hartley et al., 2007; McCary et al., 2018), which would support our results. However, because this pattern for ground beetles was not as strong as the other arthropod groups in this study, we interpret this result with caution.

Despite revealing that decreasing habitat connectivity reduces ground arthropod abundances and alters their community composition, we found that their alpha diversity (i.e., local diversity) was unaffected. We propose several reasons to explain this result. First, it is possible we did not see changes in ground arthropod alpha diversity because of the short‐term length of the experiment. Although the length of our study was long enough to detect differences in the abundance and composition of ground arthropods, it is possible that the experiment's length was too short to observe an effect on arthropod alpha diversity. Perhaps it will take several additional generations of isolation before species go extinct in those patches, thereby negatively impacting alpha diversity. An alternative explanation for the lack of alpha diversity response might simply be that the local diversity of ground arthropods is not affected by connectivity, even though the regional diversity (i.e., beta diversity) could be affected. This finding would indicate that decreasing habitat connectivity would generally have a negative influence on ground arthropod abundance and beta diversity but that the local species pool would not be affected.

In our second hypothesis, we expected less mobile arthropods to be more affected by habitat connectivity than more‐mobile arthropods. Our results strongly support this hypothesis, showing that flying arthropods were unaffected by decreasing habitat connectivity, while ground arthropods were highly affected. For example, the abundance, diversity, and community composition of flying arthropods were completely unaffected by habitat connectivity; in contrast, we detected significant differences in the abundance and composition of ground arthropods relating to habitat connectivity. Because flying arthropods can travel long distances and, therefore, operate on a larger scale than ground arthropods (Kuussaari et al., 2014), their communities could still reach isolated patches in our experiment. On the other hand, the smaller and less mobile arthropods became isolated after mowing, limiting movement in and out of the patches. To our knowledge, this is the first study to show how flying and ground arthropods differ in their metacommunity structure.

We also found evidence that decreasing habitat connectivity has the potential to alter trophic interactions of ground arthropods. Although these arthropods are generally less mobile, there is still a wide variation in mobility across taxonomic groups, particularly considering micro‐ versus macro‐detritivores. For example, micro‐detritivores (e.g., springtails and mites) are generally <2 mm in size and often limited in dispersal ability (Coleman & Hendrix, 2000), with their habitats ranging between 5 and 10 m^2^ in their lifespan (Bardgett, 2005). Alternatively, larger macro‐detritivores, such as millipedes (Diplopoda) and sow bugs (Isopoda), are incredibly mobile (Magura et al., 2008), rendering them more likely to transverse into and out of isolated patches. Such varying levels of mobility could make micro‐detritivores more susceptible to predators due to their limited ability to leave patches containing highly mobile predators, whereas macro‐detritivores might be less susceptible due to their ability to disperse more easily. Our SEM shows that predators have a strong positive correlation with micro‐detritivores, suggesting that micro‐detritivores experience higher predation pressure; macro‐detritivores showed no relationship with predators (Figure 3). This finding supports a growing body of evidence showing that predators can influence metacommunity structure (Cohen et al., 1993; Orrock et al., 2008; Polis et al., 1997).

## 5 CONCLUSIONS

Enhancing habitat connectivity has been presented as a possible solution to restore the degraded and fragmented habitats resulting from anthropogenic global change (Gawecka & Bascompte, 2021; Swan & Brown, 2017). In this study, we reveal that flying insects with greater dispersal abilities were unaffected by habitat connectivity. However, we found that decreasing habitat connectivity can negatively influence ground arthropod communities and their associated metacommunity structure, with potential implications for trophic interactions. Because of their essential roles in terrestrial ecosystem functioning and services (Bardgett & van der Putten, 2014; Coleman & Hendrix, 2000), we advocate for conservationists, restoration practitioners, and land managers to include principles of habitat connectivity for ground arthropods when designing biodiversity management programs.

## AUTHOR CONTRIBUTIONS

**Franklin Bertellotti:** Data curation (equal); project administration (lead); writing – original draft (lead); writing – review and editing (supporting). **Nathalie R. Sommer:** Data curation (supporting); methodology (supporting); supervision (supporting); writing – original draft (supporting); writing – review and editing (supporting). **Oswald J. Schmitz:** Conceptualization (equal); funding acquisition (equal); resources (lead); supervision (equal); writing – original draft (supporting); writing – review and editing (supporting). **Matthew A. McCary:** Conceptualization (equal); formal analysis (lead); funding acquisition (equal); supervision (equal); writing – original draft (equal); writing – review and editing (lead).

## Supporting information


Appendix S1

## Data Availability

Access to all data and accompanying scripts are available at Zenodo: 10.5281/zenodo.10019940.
