# Supplementary material for: Impacts of habitat connectivity on grassland arthropod metacommunity structure: A field‐based experimental test of theory
Source: Ecol Evol. 2023 Nov 7;13(11):e10686. doi: 10.1002/ece3.10686 (PMC10630154; doi:10.1002/ece3.10686)
Supplement: Supplementary file 1 — Appendix S1 [file ECE3-13-e10686-s001.docx]

**Impacts of habitat connectivity on grassland arthropod metacommunity structure: a field-based experimental test of theory**

**Appendix S1.** Experimental design and supplemental analyses.


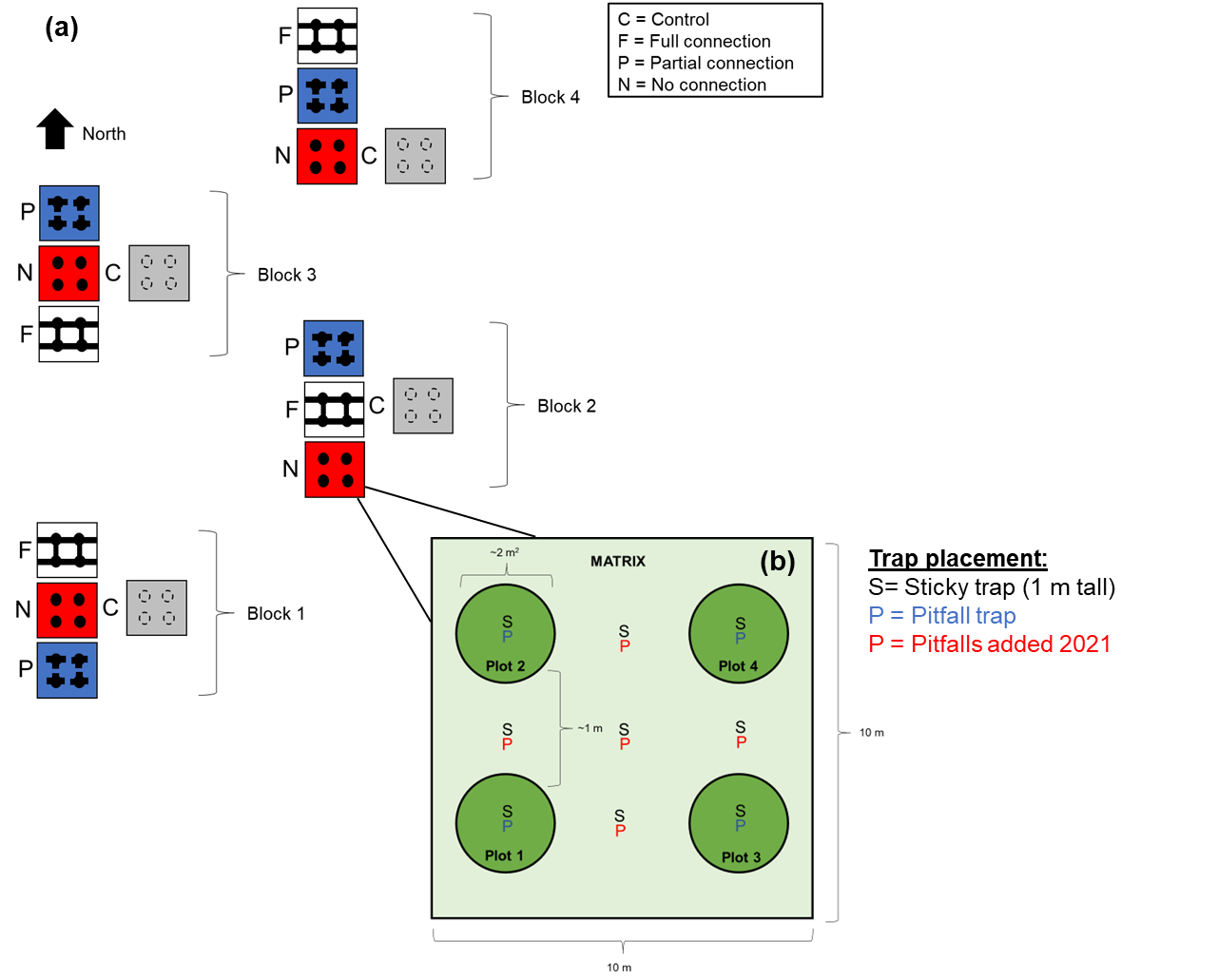


**Fig. S1**. (a) The experimental layout to test how habitat connectivity affects the metacommunity structure of ground and flying arthropods. (b) The sampling design for pitfall and sticky traps for capturing ground and flying insects, respectively. Control (un-mowed) = Gray, Full connection = Black, Partial connection = Blue, and No connection = Red. These colors mirror the color scheme shown in the main text.

**Table S1.** Statistical models (Linear mixed-effects models and PERMANOVAs) that test fixed factors of habitat connectivity and year (2020 and 2021). Bold and italicized numbers indicated marginal interactions (i.e., 0.05 < *P* ≤ 0.1). LMM = Linear mixed-effects model; PERMANOVA = Permutational Analysis of Variance.

| **Factor** | **Num df** | **Den df** | **F/Pseudo-F** | **P** |
| --- | --- | --- | --- | --- |
| *(a) Ground arthropod activity-density (LMM)* | | | | |
| Treatment | 3 | 21 | 1.33 | 0.291 |
| Year | 1 | 21 | 37.91 | <0.001 |
| Treatment x Year | 3 | 21 | 2.33 | ***0.100*** |
|  |  |  |  |  |
| *(b) Ground arthropod alpha diversity (LMM)* | | | | |
| Treatment | 3 | 24 | 0.47 | 0.700 |
| Year | 1 | 24 | 60.37 | <0.001 |
| Treatment x Year | 3 | 24 | 2.04 | 0.135 |
|  |  |  |  |  |
| *(c) Ground arthropod beta diversity (PERMANOVA)* | | | | |
| Treatment | 3 | 9 | 1.35 | 0.189 |
| Year | 1 | 3 | 24.30 | 0.014 |
| Block | 3 | 9 | 1.54 | 0.172 |
| Treatment x Year | 3 | 9 | 1.92 | ***0.094*** |
| Treatment x Block | 9 | 9 | 1.08 | 0.422 |
| Year x Block | 3 | 9 | 1.57 | 0.169 |
|  |  |  |  |  |
| *(d) Flying arthropod abundance (LMM)* | | | | |
| Treatment | 3 | 21 | 1.91 | 0.159 |
| Year | 1 | 21 | 3.19 | 0.089 |
| Treatment x Year | 3 | 21 | 0.14 | 0.936 |
|  |  |  |  |  |
| *(e) Flying arthropod alpha diversity (LMM)* | | | | |
| Treatment | 3 | 21 | 1.20 | 0.333 |
| Year | 1 | 21 | 31.85 | <0.001 |
| Treatment x Year | 3 | 21 | 0.52 | 0.673 |
|  |  |  |  |  |
| *(f) Flying arthropod beta diversity (PERMANOVA)* | | | | |
| Treatment | 3 | 9 | 1.40 | 0.204 |
| Year | 1 | 3 | 122.79 | 0.002 |
| Block | 3 | 9 | 3.97 | 0.015 |
| Treatment x Year | 3 | 9 | 0.69 | 0.652 |
| Treatment x Block | 9 | 9 | 1.80 | 0.126 |
| Year x Block | 3 | 9 | 0.97 | 0.461 |

**Table S2.** Statistical models testing for the effects of habitat connectivity on ground and flying insects in (a) 2020 and (b) 2021 separately. The column label ‘Model’ represents the specific statistical model used to investigate the relationship between habitat connectivity and associated variable of interest (i.e., abundance and diversity (alpha and beta); see main text for full details of the analysis). Bolded and italicized numbers indicate *P* < 0.05. LMM = Linear mixed-effects model; PERMANOVA = Permutational Analysis of Variance.

|  | |  |  | ***(a) 2020*** | | ***(b) 2021*** | |
| --- | --- | --- | --- | --- | --- | --- | --- |
| **Taxa** | | **Variable** | **Model** | **F value** | ***P*** | **F value** | ***P*** |
| *Ground arthropods* |  | |  |  |  |  |  |
|  | | Simpson’s Diversity | LMM | 2.69 | 0.11 | 0.73 | 0.56 |
|  | | Total abundance | LMM | 1.74 | 0.21 | 5.24 | ***0.02*** |
|  | | Composition | PERMANOVA | 1.23 | 0.27 | 2.36 | ***0.03*** |
|  | |  |  |  |  |  |  |
| *Flying arthropods* | |  |  |  |  |  |  |
|  | | Simpson’s Diversity | LMM | 1.36 | 0.32 | 0.39 | 0.76 |
|  | | Total abundance | LMM | 0.88 | 0.49 | 2.39 | 0.12 |
|  | | Composition | PERMANOVA | 1.24 | 0.30 | 1.02 | 0.46 |

**Table S3.** Permutational analysis of variance (PERMANOVA) testing for the effects of habitat connectivity on the beta diversity of ground arthropods in 2021. The PERMANOVA (9,999 permutations; Type III SS) is based on a Bray-Curtis dissimilarity distance matrix.

| **Source** | **df** | **SS** | **MS** | **Pseudo-F** | ***P*** |
| --- | --- | --- | --- | --- | --- |
| Treatment | 3 | 865.32 | 288.44 | 2.36 | 0.033 |
| Block | 3 | 839.93 | 279.98 | 2.29 | 0.036 |
| Residual | 9 | 1101.60 | 122.40 |  |  |
| Total | 15 | 2806.80 |  |  |  |
|  |  |  |  |  |  |
| **Pairwise comparisons** | **t** | ***P*** |  |  |  |
| Control, Full connection | 1.07 | 0.359 |  |  |  |
| Control, No connection | 2.07 | 0.035 |  |  |  |
| Control, Partial connection | 0.97 | 0.457 |  |  |  |
| Full connection, No connection | 1.49 | 0.110 |  |  |  |
| Full connection, Partial connection | 1.33 | 0.177 |  |  |  |
| No connection, Partial connection | 2.03 | 0.042 |  |  |  |

**
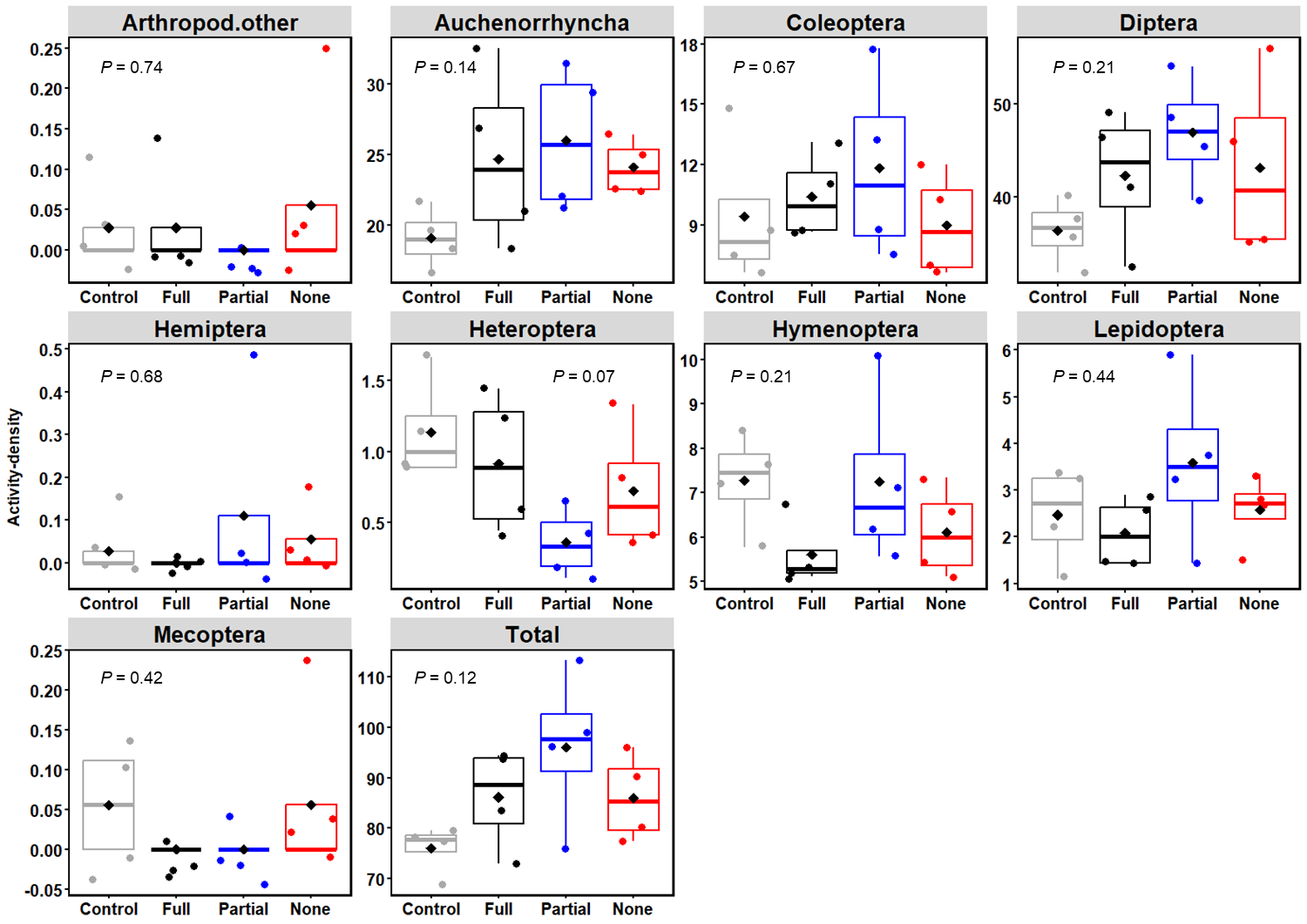
**

**Fig. S2**. Results showing the impact of habitat connectivity on flying arthropods in ***2021*.** No flying insect group had a strong relationship with habitat connectivity. The top and bottom of the boxes indicate the first and third quartiles for all boxplots; the center line denotes the median and the whiskers are 1.5 times the interquartile range. The diamond symbols reflect treatment means for 2021.


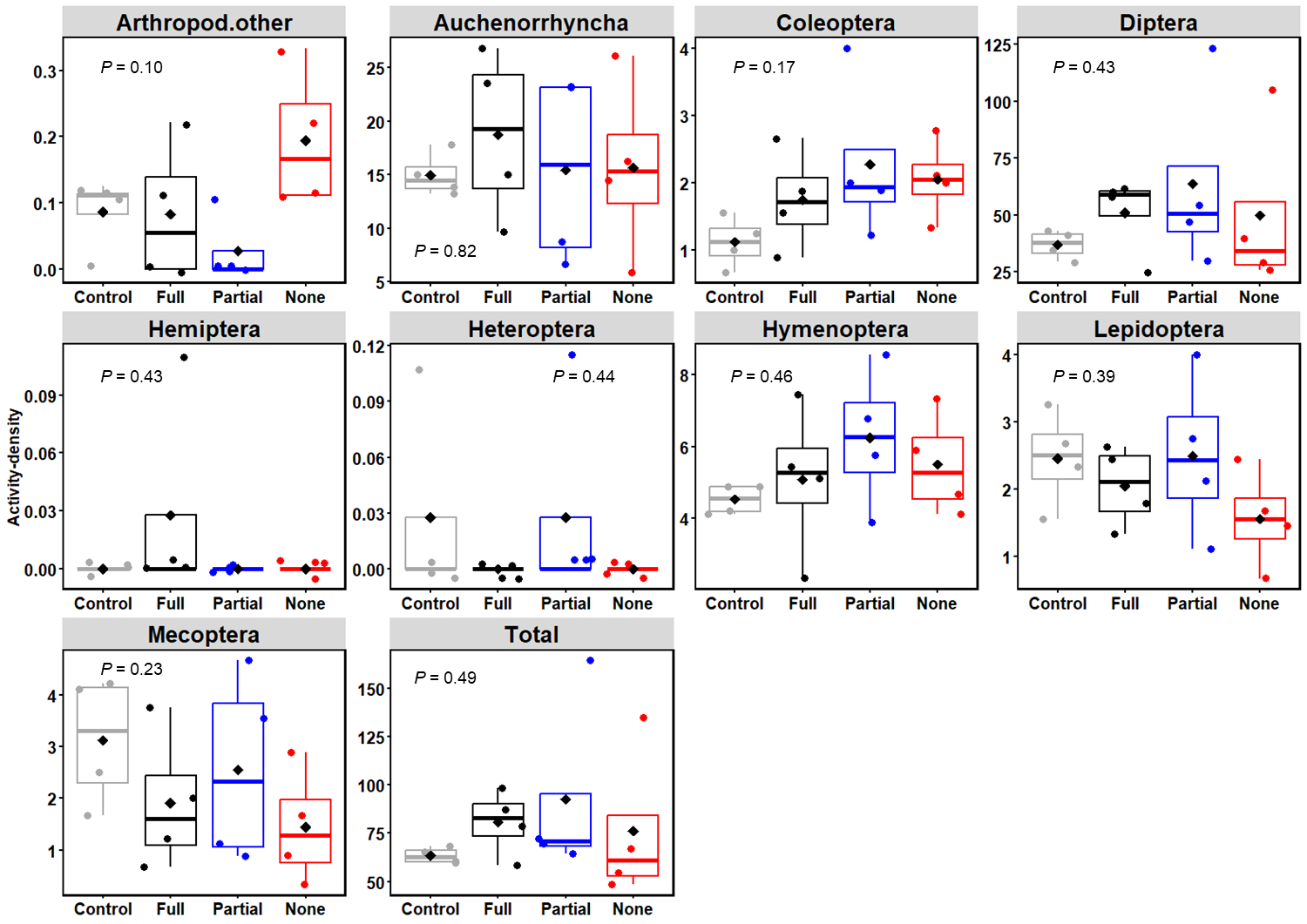


**Fig. S3**. Results showing the impact of habitat connectivity on flying arthropods in ***2020***. The top and bottom of the boxes indicate the first and third quartiles for all boxplots; the center line denotes the median and the whiskers are 1.5 times the interquartile range. The diamond symbols are treatment means for 2020.


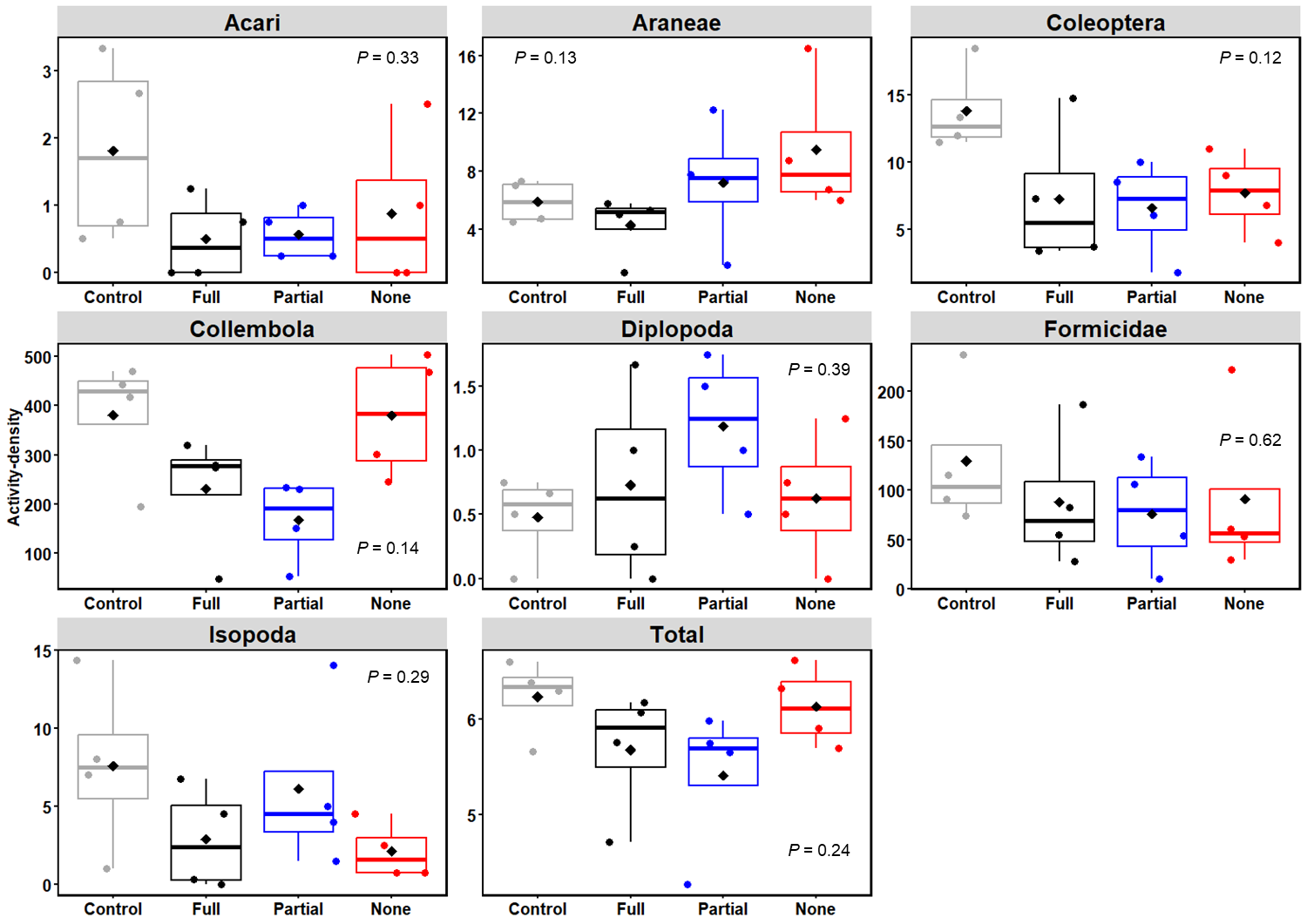


**Fig. S3.** The relationship between habitat connectivity and individual ground arthropod taxa for ***2020***. For all boxplots, the top and bottom of the boxes indicate the first and third quartiles, with the center line denoting the median; the whiskers show 1.5 times the interquartile range. The diamond symbols are treatment means for 2020.
